# Left-sided primary tumor is a favorable prognostic factor for metastatic colorectal cancer patients receiving surgery

**DOI:** 10.18632/oncotarget.18896

**Published:** 2017-06-30

**Authors:** Xiao-Fen Li, Yi-Nuo Tan, Chen-Han Zhong, Li-Zhen Zhu, Xue-Feng Fang, Jun Li, Ke-Feng Ding, Ying Yuan

**Affiliations:** ^1^ Department of Medical Oncology, The Second Affiliated Hospital, Zhejiang University School of Medicine, Hangzhou, China; ^2^ Department of Surgical Oncology, The Second Affiliated Hospital, Zhejiang University School of Medicine, Hangzhou, China; ^3^ Key Laboratory of Cancer Prevention and Intervention of Ministry of Education, The Second Affiliated Hospital, Zhejiang University School of Medicine, Hangzhou, China

**Keywords:** metastatic colorectal cancer, primary tumor location, surgery

## Abstract

**Objective:**

The role of surgery in metastatic colorectal cancer (mCRC) remains controversial. This study was performed to assess the impact of surgery on survival in metastatic colorectal cancer.

**Materials and Methods:**

Information of mCRC patients diagnosed between January 1, 2004, and December 31, 2013, was retrieved from the Surveillance, Epidemiology, and End Results Program database. Patients were classified in three groups: patients undergoing resection of both primary and distant metastatic tumors (group ‘PMTR’), patients receiving primary tumor resection alone (group ‘PTR’) and patients not undergoing any surgery (group ‘No resection’). Kaplan-Meier method and multivariate Cox proportional hazard regression analysis were applied to estimate disease specific survival time (DSS) and determine prognostic factors.

**Results:**

A total of 38,591 mCRC patients were eligible. Overall, median DSS of group ‘PMTR’ was significantly longer compared with group ‘PTR’ and group ‘No resection’ (28.0 vs 21.0 vs 11.0 months, *P* < 0.001). Stratified analysis observed that primary tumor in left-sided colorectal cancer (LCRC) was a favorable prognostic factor compared with right-sided colorectal cancer (RCRC) (median DSS of LCRC: PMTR, 34 months, PTR, 25 months, No resection, 13 months; median DSS of RCRC: PMTR, 20 months, PTR, 16 months, No resection, 8 months; *P* < 0.001). Multivariate analysis demonstrated that surgery was an independent prognostic factor for better survival (PMTR, HR = 0.403, 95% CI 0.384–0.423, *P* < 0.001; PTR, HR = 0.515, 95% CI 0.496–0.534, *P* < 0.001). Furthermore, in patients undergoing surgery, patients with younger age, female, married status, LCRC and lower CEA level were prone to receiving PMTR.

**Conclusions:**

This analysis demonstrated that surgery was an independent prognostic factor for improved survival in mCRC. Patients with LCRC had better survival than patients with RCRC after surgery.

## INTRODUCTION

The statistics of World Health Organization (WHO) have shown that colorectal cancer (CRC) is the third most common malignancy worldwide and the fourth leading cause of cancer-related deaths [1]. According to the Surveillance, Epidemiology, and End Results Program (SEER) cancer statistics, about 20% of CRC patients have distant metastases at the time of initial diagnosis, with a 5-year survival rate of 13.1% [2].

The main treatment options for metastatic CRC are surgery, systematic therapy and radiation. For patients with resectable liver and lung metastases, studies indicated that radical resection of primary and metastatic tumors have significant survival benefit [3–6]. Taking liver metastatic CRC as an example, a recent retrospective study reported a median 5-year survival rate of 38% in CRC patients with hepatic metastases who had undergone complete resection of primary tumor and liver metastases [7]. Additionally, analysis showed that metastatic CRC (mCRC) patients with solitary liver metastases had a 5-year survival rate as high as 71% [8]. Therefore, current National Comprehensive Cancer Network (NCCN) guidelines recommend complete resection of resectable liver and lung metastases in suitable mCRC patients [9, 10]. Undoubtedly, surgery is an effective and vital treatment for mCRC. However, to date, there is no clear definition to ‘suitable mCRC patients’ for surgery. It is important to find out molecular and clinical predictive factors associated with surgery in mCRC patients.

It is well known that CRC is a highly heterogeneous disease [11]. Because of different embryonic originations, left-sided CRC (LCRC) is distinguished from right-sided CRC (RCRC) in epidemiology, histology, clinical characteristics and molecular profiles [12, 13]. Some studies indicated that LCRC was a favorable prognostic factor of CRC patients’ survival [12, 14]. Recently, primary tumor location of CRC was reported to be associated with the efficacy of cetuximab, a monoclonal antibody targeting epidermal growth factor receptor (EGFR) [15–18]. But to date it is unclear whether primary tumor location is associated with the prognosis of mCRC patients who have undergone surgical resections.

We conducted this retrospective study to evaluate the impact of surgery on survival in mCRC patients, to investigate prognostic factors for better survival, to identify factors associated with surgery, and especially, to study the role of primary tumor location in the outcome of mCRC patients who underwent surgical resections.

## RESULTS

### Patient characteristics

A total of 38,591 patients newly diagnosed with mCRC from 2004 to 2013 were identified, including 20,857 males and 17,734 females. Patients younger than 70 years old accounted for 69.2% of the whole cohort. Overall, 6626(17.2%) patients underwent resection of both primary and distant metastatic tumors (group ‘PMTR’), 18,749 (48.6%) patients received PTR alone (group ‘PTR’), and 13216 (34.2%) patients did not undergo any surgery (group ‘No resection’). Metastatic sites and locoregional treatment data have not been recorded in the SEER database until the year of 2010. So we retrieved liver, lung and brain metastases information in patients diagnosed from 2010 to 2013. Patients’ demographic and pathological characteristics were summarized in Table 1.

**Table 1:** Characteristics of patients diagnosed with stage IV CRC from 2004 to 2013

Variables	PMTR (*n =* 6626)		PTR (*n =* 18749)		No resection (*n =* 13216)	
	*n*	%	*n*	%	*n*	%
**Age**						
< = 70 years old	4974	75.1	12784	68.2	8966	67.8
> 70 years old	1652	24.9	5965	31.8	4250	32.2
**Gender**						
Female	3527	53.2	8585	45.8	5622	42.5
Male	3099	46.8	10164	54.2	7594	57.5
**Marital status**						
Married	3813	57.5	10332	55.1	6309	47.7
Single/Divorced/Separated/Widowed	2585	39.0	7787	41.5	6277	47.5
Unknown	228	3.5	630	3.4	630	4.8
**Primary tumor site**						
RCRC	2778	41.9	8433	45.0	3971	30.0
LCRC	3848	58.1	10316	55.0	9245	70.0
**Grade**						
Well-differentiated	239	3.6	756	4.0	593	4.5
Moderate-differentiated	4196	63.3	11709	62.5	6062	45.9
Poor-differentiated	1651	24.9	5004	26.7	1922	14.5
Undifferentiated	247	3.7	527	2.8	107	0.8
Unknown	293	4.4	753	4.0	4532	34.3
**CEA**						
Positive/outside reference range	3588	54.2	9974	53.2	8332	63.0
Negative/within reference range	1105	16.7	2684	14.3	1032	7.8
Unknown	1933	29.2	6091	32.5	3852	29.1
T-stage*						
T1	103	1.6	403	2.1	2469	18.7
T2	178	2.7	541	2.9	178	1.3
T3	3694	55.8	11105	59.2	1864	14.1
T4	2532	38.2	6367	34.0	2108	16.0
Tx	119	1.8	333	1.8	6597	49.9
N-stage*						
0	1180	17.8	3412	18.2	5457	41.3
1	2354	35.5	6169	32.9	3259	24.7
2	3003	45.3	8884	47.4	487	3.7
Nx	89	1.3	284	1.5	4013	30.4
**Liver metastasis**						
Positive	1781	26.9	4810	25.7	4880	36.9
Negative	793	12.0	1809	9.6	1200	9.1
Unknown	4052	61.2	12130	64.7	7136	54.0
**Lung metastasis**						
Positive	280	4.2	1246	6.6	2149	16.3
Negative	2271	34.3	5287	28.2	3785	28.6
Unknown	4075	61.5	12216	65.2	7282	55.1
**Brain metastasis**						
Positive	31	0.5	50	0.3	81	0.6
Negative	2506	37.8	6462	34.5	5827	44.1
Unknown	4089	61.7	12237	65.3	7308	55.3

Note: *In the ‘No resection’ group, the reported T and N stage have been clinically evaluated based on imaging techniques. Abbreviations: CRC, colorectal cancer; PMTR, resection of both primary and distant metastatic tumors; PTR, primary tumor resection; CEA, carcinoembryonic antigen.

### Survival analyses for different groups

**Figure 1:**
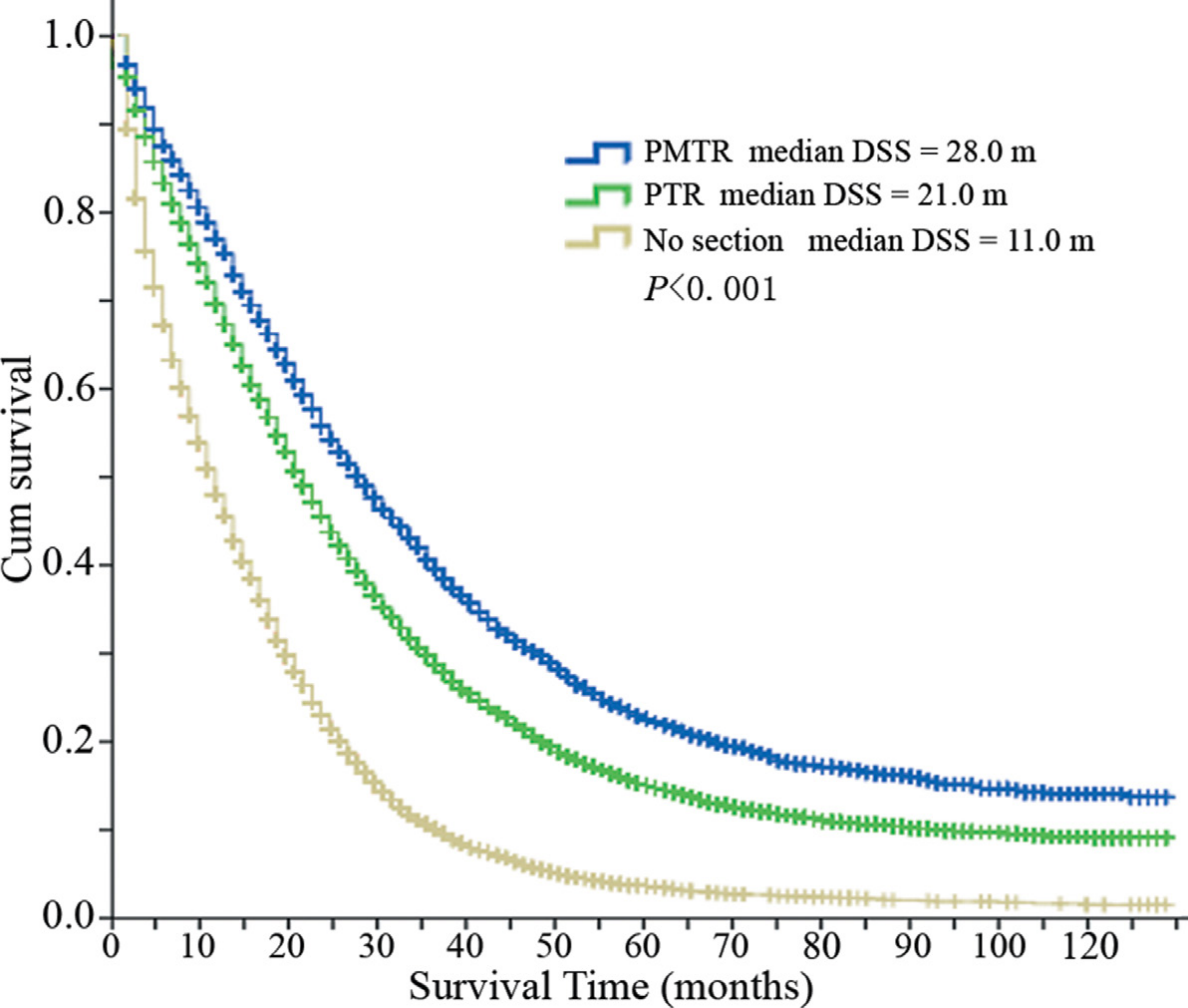
Disease specific survival curves of different groups Median DSS of group ‘PMTR’ = 28.0 months, median DSS of group ‘PTR’ = 21.0 months, and median DSS of group ‘No resection’ = 11.0 months, *P <* 0.001. Abbreviations: DSS, disease specific survival; PMTR, resection of both primary and distant metastatic tumors; PTR, primary tumor resection.

**Figure 2:**
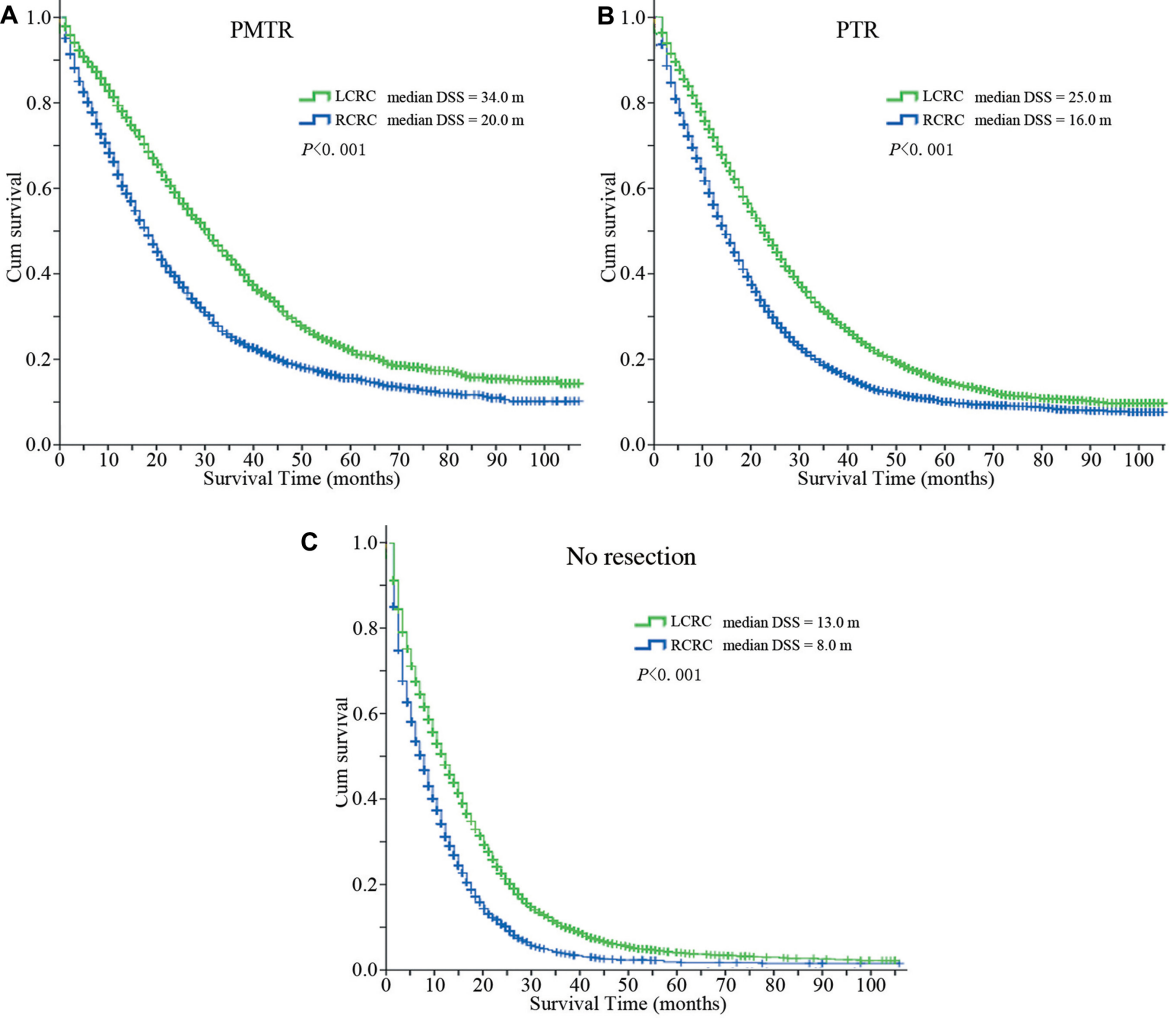
Disease specific survival curves of patients with different primary tumor locations (**A**) Disease specific survival curves of patients with different primary tumor locations in group ‘PMTR’ (median DSS, LCRC = 34.0 months, RCRC = 20.0 months, *P <* 0.001). (**B**) Disease specific survival curves of patients with different primary tumor locations in group ‘PTR’ (median DSS, LCRC = 25.0 months, RCRC = 16.0 months, *P <* 0.001). (**C**) Disease specific survival curves of patients with different primary tumor locations in group ‘No resection’ (median DSS, LCRC = 13.0 months, RCRC = 8.0 months, *P <* 0.001). Abbreviations: DSS, disease specific survival; PMTR, resection of both primary and distant metastatic tumors; PTR, primary tumor resection; LCRC, left-sided colorectal cancer; RCRC, right-sided colorectal cancer.

In total, Kaplan-Meier analysis and log-rank (Mantel-Cox) test showed that median disease specific survival (DSS) of group ‘PMTR’ was significantly longer compared with group ‘PTR’ and group ‘No resection’ (28.0 vs 21.0 vs 11.0 months, *P <* 0.001, Table 2, Figure 1). We could see that median DSS of patients receiving surgery was significantly longer than that of patients who did not undergo any surgery (Figure 1). Stratified analysis observed that LCRC was a favorable prognostic factor compared with RCRC (median DSS of LCRC: PMTR, 34 months, PTR, 25 months, No resection, 13 months; median DSS of RCRC: PMTR, 20 months, PTR, 16 months, No resection, 8 months; *P <* 0.001, Figure 2). In group ‘PMTR’, median DSS of patients with LCRC was 14 months longer than that of patients with RCRC (Figure 2A).

**Table 2:** Multivariate analysis for prognostic factors associated with disease specific survival

Variable	HR	95% CI	*P*
**Age**			
> 70 years old	1		
< = 70 years old	0.626	0.604–0.648	< 0.001
**Gender**			
Male	1		
Female	0.995	0.979–1.011	0.508
**Marital status**			
Married	1		
Single/Divorced/Separated/Widowed	1.072	1.055–1.090	< 0.001
**Primary tumor site**			
LCRC	1		
RCRC	1.374	1.330–1.421	< 0.001
**Grade**			
Undifferentiated	1		
Well-differentiated	0.756	0.711–0.804	< 0.001
Moderate-differentiated	0.822	0.794–0.851	< 0.001
Poor-differentiated	1.254	1.207–1.303	< 0.001
**CEA**			
Positive/outside reference range	1		
Negative/within reference range	0.649	0.622–0.677	< 0.001
**Surgery**			
No resection	1		
PMTR	0.403	0.384–0.423	< 0.001
PTR	0.515	0.496–0.534	< 0.001

Abbreviations: HR, hazard ratio; CI, confidence interval; PMTR, resection of both primary and distant metastatic tumors; PTR, primary tumor resection; CEA, carcinoembryonic antigen.

**Figure 3:**
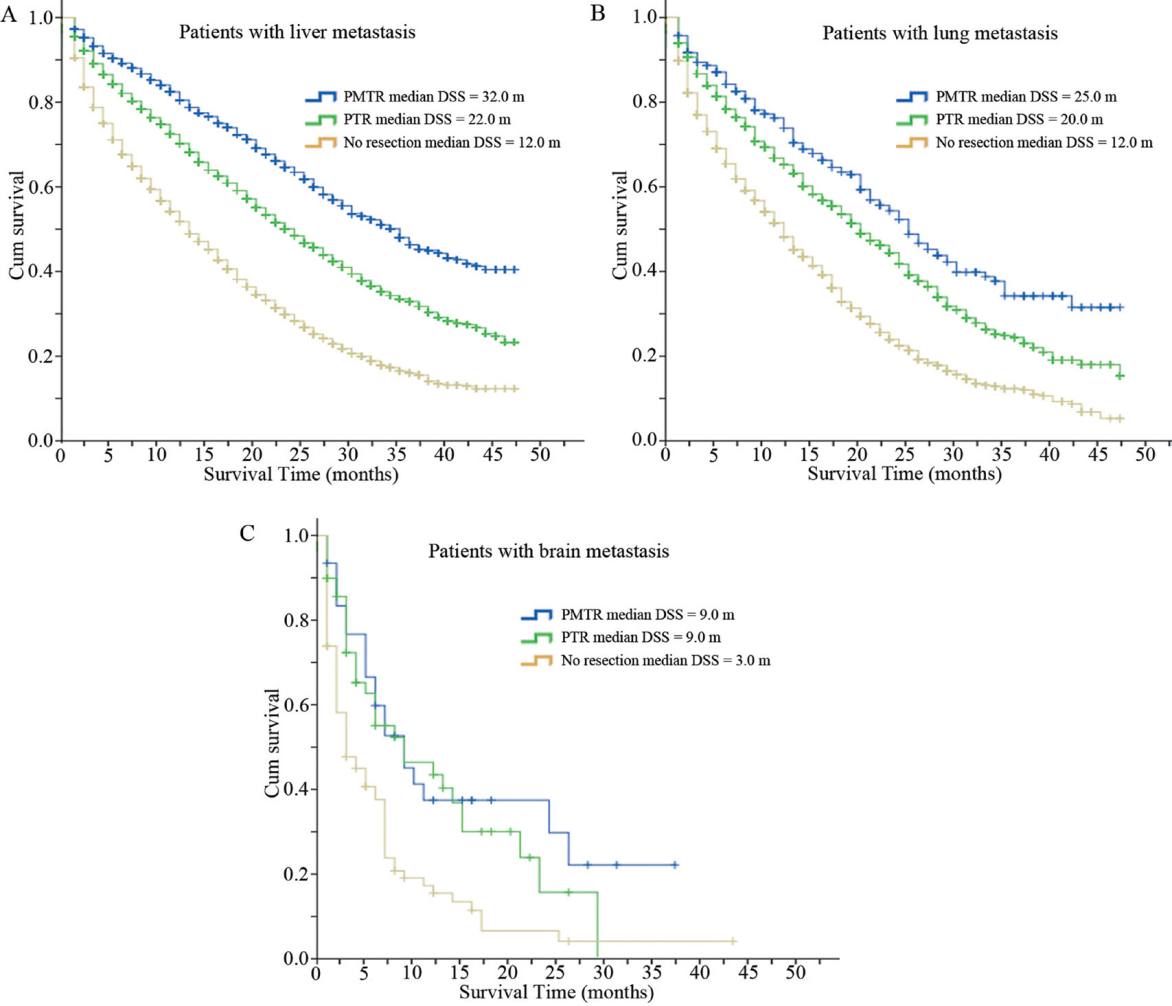
Disease specific survival curves of patients with different sites of metastasis (**A**) Disease specific survival curves of patients with liver metastasis (PMTR, 32 months, PTR, 22 months, No resection, 12 months; *P <* 0.001). (**B**) Disease specific survival curves of patients with lung metastasis (PMTR, 25 months, PTR, 20 months, No resection, 12 months; *P <* 0.01). (**C**) Disease specific survival curves of patients with brain metastasis (PMTR vs PTR, 9 months vs 9 months, *P* = 0.486; PMTR vs No resection, 9 months vs 3 months, *P* = 0.002). Abbreviations: DSS, disease specific survival; PMTR, resection of both primary and distant metastatic tumors; PTR, primary tumor resection.

Metastatic sites in liver, lung and brain data of patients diagnosed from 2010 to 2013 are available in SEER database. Therefore we further analyzed survival of patients with different sites of metastasis. The results showed that in all the 11,471 patients with liver metastasis, 1,781 (15.5%) patients received PMTR, 4,810 (41.9%) patients underwent PTR, and 4,880 (42.6%) patients were classified to group ‘No resection’. Kaplan-Meier analysis found that in patients with liver metastasis, median DSS of group ‘PMTR’ was significantly longer than the other two groups (PMTR, 32 months, PTR, 22 months, No resection, 12 months; *P <* 0.001, Figure 3A). Similar findings were obtained in patients with lung metastasis (PMTR, 25 months, PTR, 20 months, No resection, 12 months; *P <* 0.01, Figure 3B). However, in patients with brain metastasis, group ‘PMTR’ had the same survival time with group ‘PTR’, but longer than group ‘No resection’ (PMTR vs PTR, 9 months vs 9 months, *P* = 0.486; PMTR vs No resection, 9 months vs 3 months, *P* = 0.002, Figure 3C).

### Prognostic factors for survival

Multivariate Cox proportional hazard regression analysis identified some independent factors associated with survival. Improved survival or lower death risk was associated with surgery, younger age, LCRC, well-differentiated tumor, lower CEA level, married status, etc. (Table 2). Surgery was an independent prognostic factor for metastatic CRC patients (PMTR, HR = 0.403, 95% CI 0.384–0.423, *P <* 0.001; PTR, HR = 0.515, 95% CI 0.496–0.534, *P <* 0.001). In other words, patients who underwent surgery showed significantly decreased death risk compared with those not receiving any surgery. Because the information of metastatic sites was not comprehensive, we did not include sites of metastasis when analyzing prognostic factors.

### Factors associated with surgery

Results of logistic regression analysis demonstrated that patients with younger age, female, married status, RCRC and lower CEA level were more likely to undergo surgery (Table 3). Furthermore, in patients receiving surgery (PMTR or PTR), patients with younger age, female, married status, LCRC and lower CEA level were prone to receiving PMTR (Table 4), while patients with older age, male, unmarried status (single/divorced/ separated/widowed), RCRC and higher CEA level were more likely to receive PTR.

**Table 3:** Logistic regression analysis of factors associated with surgery

Variables	OR	95% CI	*P*
**Age**			
> 70 years old	1		
< = 70 years old	1.124	1.050–1.204	0.001
**Gender**			
Male	1		
Female	1.247	1.172–1.328	< 0.001
**Marital status**			
Married	1		
Single/Divorced/Separated/Widowed	0.730	0.686–0.776	< 0.001
**Primary tumor site**			
LCRC	1		
RCRC	1.967	1.840–2.103	< 0.001
**Grade**			
Undifferentiated	1		
Well-differentiated	0.229	0.172–0.307	< 0.001
Moderate-differentiated	0.381	0.293–0.496	< 0.001
Poor-differentiated	0.431	0.329–0.564	< 0.001
**CEA**			
Positive/outside reference range	1		
Negative/within reference range	1.954	1.790–2.134	< 0.001

Abbreviations: PMTR, resection of both primary and distant metastatic tumors; OR, odds ratio; CI, confidence interval; CEA, carcinoembryonic antigen.

**Table 4:** Logistic regression analysis of factors associated with PMTR

Variables	OR	95% CI	*P*
**Age**			
> 70 years old	1		
< = 70 years old	1.385	1.277–1.502	< 0.001
**Gender**			
Male	1		
Female	1.381	1.286–1.482	< 0.001
**Marital status**			
Married	1		
Single/Divorced/Separated/Widowed	0.884	0.823–0.950	0.001
**Primary tumor site**			
LCRC	1		
RCRC	0.866	0.806–0.931	< 0.001
**Grade**			
Undifferentiated	1		
Well-differentiated	0.650	0.500–0.846	0.001
Moderate-differentiated	0.810	0.668–0.983	0.032
Poor-differentiated	0.747	0.612–0.912	0.004
**CEA**			
Positive/outside reference range	1		
Negative/within reference range	1.170	1.076–1.271	< 0.001

Abbreviations: PMTR, resection of both primary and distant metastatic tumors; OR, odds ratio; CI, confidence interval; CEA, carcinoembryonic antigen.

## DISCUSSION

This retrospective cohort study indicated that median DSS of patients receiving surgery, especially those who received both metastasectomy and PTR, was significantly longer than that of patients who did not undergo any surgery (28.0 vs 21.0 vs 11.0 months, *P <* 0.001). Specifically, patients of LCRC had better survival compared with patients of RCRC, especially in surgically resected setting (median DSS of LCRC: PMTR, 34 months, PTR, 25 months, No resection, 13 months; median DSS of RCRC: PMTR, 20 months, PTR, 16 months, No resection, 8 months; *P <* 0.001). Furthermore, patients with liver and lung metastasis benefited from PMTR if it was feasible, but in patients with brain metastasis, group ‘PMTR’ had similar survival to group ‘PTR’. Multivariate analysis demonstrated that surgery was an independent prognostic factor for improved survival or decreased death risk (PMTR, HR = 0.403, 95% CI 0.384–0.423, *P <* 0.001; PTR, HR = 0.515, 95% CI 0.496–0.534, *P <* 0.001). Furthermore, logistic regression analysis revealed that patients with younger age, female, married status, LCRC and lower CEA level were more likely to undergo PMTR.

As we mentioned above in the introduction section, studies demonstrated that hepatic and pulmonary metastasectomy had significant survival benefits to suitable patients [3–8]. With radical resection of metastatic and primary tumors, 5-year survival rate of liver metastatic CRC patients improved from 13.1% to 38% [2, 7]. Unfortunately, over 80% of patients with stage IV disease present with unresectable metastases [19]. In those cases, the role of palliative PTR remains controversial. A recent retrospective study in the USA showed that the annual rate of PTR decreased from 74.5% in 1988 to 57.4% in 2010 (*P <* 0.001), while median relative survival rate improved from 8.6% in 1988 to 17.8% in 2009 (*P <* 0.001) [20]. Yun et al. conducted a study to investigate the prognostic role of PTR in asymptomatic unresectable mCRC patients. Their results showed that palliative PTR was not associated with better survival compared with nonresection after propensity score matching (5-year survival rate, 4.9% vs 3.5%, *P* = 0.27) [21]. However, some studies indicated that PTR was beneficial to the survival of mCRC patients [22–27]. A study including 834 asymptomatic or minimally symptomatic patients diagnosed with stage IV CRC performed by Ahmed et al. showed that patients receiving palliative PTR had improved overall survival compared with non-resection group (19.7 vs 8.4 months, *P <* 0.0001). And PTR was an independent prognostic factor correlated with superior survival (HR = 0.47, 95% CI 0.39–0.57) [24]. In some studies about the value of PTR, the symptoms of primary tumor were unknown [22, 23, 25]. A pooled analysis of individual data from four randomized trials conducted by Faron et al. revealed that PTR was independently associated with better OS (HR = 0.63, 95% CI 0.53–0.75; *P <* 0.001) in mCRC patients with unresectable metastases [22]. Tarantino et al. conducted a retrospective cohort study based on SEER registry, and found that PTR was associated with a significantly improved OS (*n =* 37793, HR = 0.40, 95% CI 0.39–0.42, *P <* 0.001) and cancer-specific survival (HR = 0.39, 95% CI 0.38–0.40, *P <* 0.001) in incurable stage IV CRC patients [25]. Similar to those reported studies, our results also demonstrated the survival benefit of surgeries including PMTR and PTR in mCRC patients.

Furthermore, our study revealed that LCRC was a favorable prognostic factor compared with RCRC. In PMTR group, patients with LCRC had 14 months longer DSS than patients with RCRC. In 2008, Meguid et al. [14] conducted a population-based study to compare the survival of right- and left-sided colon cancers. Their results showed that left-sided colon cancers had a better prognosis than right-sided colon cancers (median OS, 89 months vs 78 months, *P <* 0.001). Recently, some studies demonstrated that LCRC was a good predictive factor for cetuximab efficacy [15–18]. In 2014, Von Einem and his colleagues [15] reanalyzed the data of AIO KRK-0104 trial to investigate the impact of primary tumor site on efficacy of cetuximab in mCRC patients. Their results suggested that in *KRAS* wild-type patients treated with cetuximab and chemotherapy, left-sided primary tumors were associated with significantly longer OS compared to right-sided primary tumors (26.3 vs 14.8 months, *P* = 0.016, HR = 0.63). In 2016, Chen et al. [18] published the results of a nationwide cohort study, which rerolled 969 mCRC patients with *KRAS* wild type receiving cetuximab as third-line treatment. Similarly, their results demonstrated that left-sided primary tumor was a favorable predictor of improved cetuximab efficacy in *KRAS* wild type patients (median OS of patients with left-sided primary tumors and right-sided primary tumors, 12.2 vs 8.07 months, *P <* 0.001). Up to now, the reason for the different survival and clinical behavior between LCRC and RCRC remains unclear. In embryonic origination, LCRC is developed from hindgut, while RCRC is developed from midgut. Therefore it is speculated that differences in embryonic origin and fecal exposure are closely related to the different biological behaviors of LCRC and RCRC. Some studies have revealed that gene expression of LCRC is vastly different from that of RCRC [13, 28]. For example, the frequencies of microsatellite-instability high (MSI-H) phenotype and *KRAS* mutant type in RCRC are higher than those in LCRC. Furthermore, the tumorigenesis mechanism also varies according to different primary site. Comprehensive study on the exact mechanisms of different clinical and biological behaviors in LCRC and RCRC is warranted, which will do great help to personalized and precise medicine. Our study showed that in PMTR group, median DSS of patients with LCRC was 14 months longer than that of patients with RCRC (*P <* 0.0001), which indicated that doctors might take more aggressive approach when treating mCRC patients with LCRC.

There are some advantages in our study. Firstly, the large sample size of 38,591 can help reducing sampling error. Besides, this study is based on a real world population, which facilitates improving reliability. However, there exist some limitations too. First of all, the intrinsic methodological limitations of retrospective studies exist in our study as well, including selection bias, potential confounders, etc. Second, the SEER database does not include information about patients’ performance status, comorbidities, chemotherapy, and all the sites and numbers of metastases, etc. Similarly, we could not know whether the primary tumor was asymptomatic or not, which may influence the choice of surgery. In addition, since T-stages of patients in group ‘No resection’ were inaccurate/unknown, most of which were documented as ‘Tx’, we could not perform stratified analysis by T-stage in group ‘No resection’. And it was the same with N-stage. Finally, the detailed information about surgery, such as the specific site of metastasectomy and surgical margins is not available in the SEER database, which may affect patients’ survival and results of our analysis.

## MATERIALS AND METHODS

### Patient selection

The data source of this retrospective cohort study was from the SEER database (SEER*Stat 8.3.2) [29]. The SEER Program of the National Cancer Institute collects data on cancer cases from 18 cancer registries in the United States (US), covering approximately 28% of the US population. Collected data in the SEER Program includes patient demographics, primary tumor site, tumor histology and stage at initial diagnosis, surgery, radiotherapy, death causes (due to this cancer or not) and survival time. Chemotherapy and personal identifying information are not included. The specific sites of metastasectomy and locoregional treatment data have not been recorded until the year of 2010. In this study, we retrieved liver, lung and brain metastases information in patients diagnosed from 2010 to 2013.

Patients with histologically confirmed stage IV CRC diagnosed between January 1, 2004, and December 31, 2013, were eligible in our study. All the patients were newly diagnosed with stage IV CRC. In other words, there were no recurrent cases. Histological types were restricted to adenocarcinoma (ICD-O-3, 8010,8020-8022, 8140-8141, 8144-8145, 8210-8211, 8220-8221, 8230-8231, 8260-8263), mucinous adenocarcinoma (ICD-O-3, 8472, 8473, 8480, 8481) and signet ring cell carcinoma (ICD-O-3, 8490). The code of primary tumor surgery is RX summ-Surg Prim site (1998+) > = 30 and < = 80. The code of no surgery on primary tumor is RX summ-Surg Prim site (1998+) = 0. The code of metastatic tumor surgery is RX Summ—Surg Oth Reg/Dis (2003+)! = None; diagnosed at autopsy. And the code of no surgery on metastatic tumor is RX Summ—Surg Oth Reg/Dis (2003+) = None; diagnosed at autopsy. Group ‘PMTR’ received both primary and metastatic tumors surgeries; group ‘PTR’ received only primary tumor surgery; and group ‘No resection’ did not undergo any surgery. Exclusion criteria included age younger than 18 years or older than 90 years, survival time of less than 1 month after confirmed diagnosis, CRC not the first and only diagnosis of malignant tumor, metastasectomy without PTR, occult CRC (no evidence of primary tumor). The remaining patients were grouped in three subsets: patients who underwent resection of both primary and distant metastatic tumors (group ‘PMTR’), patients that received PTR alone (group ‘PTR’) and patients that did not undergo any surgery (group ‘No resection’). In addition, primary tumors located in rectum, sigma, descending colon and the splenic flexure were defined as left-sided colorectal cancer (LCRC), while primary tumors originating from cecum to the distal part of the transverse colon were categorized as right-sided colorectal cancer (RCRC).

### Statistical analysis

Median disease specific survival time (DSS) was estimated with Kaplan-Meier method. Log-rank test was applied to compare survival time of different groups and a multivariate Cox proportional hazard regression model was established to determine the relationship between survival and other factors such as age, sex, marital status, surgery, primary tumor location, differentiation grade, CEA (carcino embryonie antigen) level, et al. Logistic regression analysis was performed to identify factors associated with surgery. Statistical tests were two sided and *P <* 0.05 was considered statistically significant. SPSS Statistics 20.0 (IBM, Armonk, NY, USA) was used to perform statistical analysis.

## Abbreviations

WHO: World Health Organization
CRC: colorectal cancer
mCRC: metastatic colorectal cancer
SEER: Surveillance Epidemiologyand End Results Program
DSS: disease specific survival
OS: overall survival
PMTR: resection of both primary and distant metastatic tumors
PTR: primary tumor resection
LCRC: left-sided colorectal cancer
RCRC: right-sided colorectal cancer
CEA: carcino embryonie antigen
HR: hazard ratio
CI: confidence interval
OR: odds ratio
MSI-H: microsatellite-instability high phenotype
